# Chest Radiographs for Pediatric TB Diagnosis: Interrater Agreement and Utility

**DOI:** 10.1155/2014/291841

**Published:** 2014-08-17

**Authors:** G. Kaguthi, V. Nduba, J. Nyokabi, F. Onchiri, R. Gie, M. Borgdorff

**Affiliations:** ^1^Kenya Medical Research Institute, Centre for Respiratory Diseases Research (CRDR), Kisumu, Nairobi 40100, Kenya; ^2^Academic Medical Centre (AMC), University of Amsterdam, Meibergdreef 9, 1105 AZ Amsterdam, The Netherlands; ^3^Africa Teleradiology, Nairobi 00200, Kenya; ^4^School of Public Health, University of Washington, Seattle, WA 98104, USA; ^5^Department of Pediatrics and Child Health, Faculty of Medicine and Health Sciences, Stellenbosch University, Cape Town, South Africa; ^6^Public Health Service of Amsterdam, Department of Infectious Diseases, 1018 WT Amsterdam, The Netherlands

## Abstract

The chest radiograph (CXR) is considered a key diagnostic tool for pediatric tuberculosis (TB) in clinical management and endpoint determination in TB vaccine trials. We set out to compare interrater agreement for TB diagnosis in western Kenya. A pediatric pulmonologist and radiologist (experts), a medical officer (M.O), and four clinical officers (C.Os) with basic training in pediatric CXR reading blindly assessed CXRs of infants who were TB suspects in a cohort study. C.Os had access to clinical findings for patient management. Weighted kappa scores summarized interrater agreement on lymphadenopathy and abnormalities consistent with TB. Sensitivity and specificity of raters were determined using microbiologically confirmed TB as the gold standard (*n* = 8). A total of 691 radiographs were reviewed. Agreement on abnormalities consistent with TB was poor; *k* = 0.14 (95% CI: 0.10–0.18) and on lymphadenopathy moderate *k* = 0.26 (95% CI: 0.18–0.36). M.O [75% (95% CI: 34.9%–96.8%)] and C.Os [63% (95% CI: 24.5%–91.5%)] had high sensitivity for culture confirmed TB. TB vaccine trials utilizing expert agreement on CXR as a nonmicrobiologically confirmed endpoint will have reduced specificity and will underestimate vaccine efficacy. C.Os detected many of the bacteriologically confirmed cases; however, this must be interpreted cautiously as they were unblinded to clinical features.

## 1. Introduction

Pediatric TB remains a challenging disease to diagnose despite advances in molecular techniques in mycobacterial identification and antigen based tests for latent TB infection [1]. Classical TB symptoms are nonspecific [2] and more so in settings with high HIV prevalence and malnutrition. Atypical presentation with acute severe pneumonia in young children has been observed [3]. Childhood TB is characterised by paucibacillary disease and microbiological confirmation is only possible in <50% of pediatric cases [1]. Chest imaging is therefore of great importance in identifying smear negative, culture negative TB. Among adults with suspected TB, several clearly defined chest radiograph features have been identified as having high interrater reliability and correlation with culture positive TB [4]. Unfortunately, similar data among infants has been limited. Lymphadenopathy is the hallmark of primary TB [5]. However, it is frequently missed due to inadequate sensitivity of the chest radiograph [6]. Chest CT scan (CT) has been considered the gold standard for detecting mediastinal lymphadenopathy, detecting up to 60% more lymphadenopathy in children with normal chest radiographs [7]. Despite this, use of CT has been limited in infant TB vaccine trials, which are set up to detect every TB endpoint so as to demonstrate efficacy. Reasons include the modest agreement on lymphadenopathy on CT [7], cost limitations, and the reluctance to use high dose ionizing radiation in young children. Thus, the chest radiograph is the mainstay of radiological diagnosis and is frequently the only tool available.

There are a limited number of studies that have described interrater agreement on chest radiograph for TB diagnosis [8, 9]. Existing studies had small sample sizes, were drawn from hospitalized children, and compared agreement only among experienced and highly trained raters; and most importantly, they focused entirely on presence of lymphadenopathy as a marker of TB. While lymphadenopathy is a key feature for diagnosing childhood TB, other radiological features also contribute to the diagnosis [5, 6].

We conducted the study in Siaya County, western Kenya, which has a high burden of both tuberculosis and HIV [10, 11]. The objective was to determine interrater agreement on any abnormality on chest radiograph and agreement on abnormalities consistent with TB among experienced and inexperienced raters. We also aimed to compare the raters' sensitivity and specificity against microbiologically confirmed TB in young children.

## 2. Study Population and Methods

### 2.1. Study Setting

The study was conducted in Siaya County, western Kenya, from June 2009 to December 2011. TB diagnosis is by the Keith Edward Score Chart [12] which assigns a score to suggestive pulmonary and extrapulmonary signs/symptoms of TB. Children who score ≥7 as well as those who score <7 but have an abnormal chest radiograph are treated for TB.

A total of 2900 BCG vaccinated infants, aged zero to six weeks and weighing at least 1700 g, were enrolled and followed up for 12–24 months to determine TB incidence. TB suspects were identified through four monthly scheduled visits and sick visits as well as by review of TB case records for contact tracing. Suspect criteria included a history of contact and/or suggestive signs and symptoms of TB and/or protocol defined hospitalization history, for example, for severe pneumonia.

### 2.2. Clinical and Laboratory Investigations

TB suspects were admitted into a case verification ward (CVW) for collection of two serial induced sputa specimens, two serial early morning gastric aspirates, DNA PCR HIV (HIV Qual test 48, Roche Molecular Systems Inc, Switzerland) testing, and Rapid HIV tests for those aged less than and greater than 18 months, respectively. Tuberculin skin testing was also done. Digital anteroposterior (AP) and lateral chest radiographs were taken at admission and images were written onto CD-ROMs.

The CD-ROMs had Digital Imaging and Communication in Medicine (DICOM) software (Phillips) that was used to view images. Readers could change the luminance of the grayscale display as well as the magnification.

### 2.3. Definitions and Classifications

Microbiologically confirmed TB (definite TB) was M. tuberculosis identified by Xpert MTB/RIF or speciated with either Capilia (FIND and Tauns co. Ltd) or GenoType assay (Hain Diagnostika, Nehren, Germany) after positive sputum culture. Probable TB was a case started on anti-TB treatment based on Keith Edward Score Chart and/or a CXR consistent with TB.

### 2.4. Raters and Training

There were four sets of raters: a radiologist and a pediatric pulmonologist (expert readers), a medical officer (M.O), and four clinical officers (C.Os). The C.Os reviewed the images, while the suspects were admitted to the CVW; all other raters read the images after close of the study and were blinded to the clinical history. All raters were trained on using the electronic interface to enter readings. The clinical and medical officer(s) were trained on reading and identifying TB on pediatric radiographs prior to the start of the study.

A chest radiograph reading form developed during a consensus meeting of TB vaccine sites (Cape Town, December 2008) was used. An electronic version was developed to standardize reporting and minimize ambivalence in the diagnosis.

The technical quality of images was assessed prior to reading. Indicators included adequacy of collimation (visibility of the lung apices, costophrenic angles with nothing obscuring the lung fields), adequate exposure, the number of visible intervertebral spaces, adequate inspiration, by counting six anterior ribs, and absence of rotation by comparing the length of the same rib on the left and right hemidiaphragms. Thereafter, the reader classified the chest radiograph quality as optimal or suboptimal or unreadable. “Unreadable” radiographs were counted as suboptimal but were still read.

Raters then systematically reviewed the images for airway narrowing, left tracheal deviation, lymphadenopathy, airway opacities, calcification, and pleural effusion. The pathology items were scored individually as present, absent, and equivocal. Final assessment of the image was normal radiograph, abnormal TB unlikely, and abnormal TB likely. Radiological signs suggestive of TB were miliary picture, airway narrowing or tracheal deviation to the left, presence of hilar, paratracheal, subcarinal, or other lymphadenopathy, evidence of calcification, cavitation, pleural effusion, or thickening.

### 2.5. Data Collection and Analysis

Data quality was assured by edit, logic, and validation checks built onto the data entry interface. Data cleaning was also conducted, and an audit trail of changes was maintained. Data was saved onto an SQL database and analysed using SAS 9.0 (SAS Institute Inc, Cary, NC, USA). To quantify degree of agreement in TB diagnosis among the raters, we estimated the individual kappas for each rating category as well as a generalized (multiple rater) chance-corrected kappa statistic (a multirater measure of agreement), which is an extension of Cohen's kappa for assessing reliability or proportion of agreement for multiple raters. The overall/generalized kappa measures agreement across all categories.

Kappa scores were interpreted as follows: poor 0.01–0.20, moderate 0.21–0.40, fair 0.41–0.60, good 0.61–0.80, or excellent 0.81–1.0 [13]. We compared agreement on any abnormality on chest radiograph as well as agreement on abnormalities consistent with TB. For comparability with previously conducted pediatric chest radiographs studies, we examined the agreement on lymphadenopathy on AP and lateral images. Sensitivity and specificity of readers' diagnosis to definite TB were also calculated. To examine each rater's propensity to place a radiograph in a certain category and thus elucidate patterns of agreement, McNemar's test for 2 × 2 tables and Bowker's test of symmetry were applied for tables with more than two categories. These methods compare marginal frequencies of each rater and test for statistically significant differences (tests of marginal homogeneity), where *P* < 0.05; marginal heterogeneity exists, that is, differing propensity to rate a category.

The study was reviewed and approved by the Kenya Medical Research Institute Ethics Review Committee (KEMRI-ERC) SSC 1465. Written informed consent was obtained from parents and guardians before study entry and for CVW investigations.

## 3. Results

**Figure 1:**
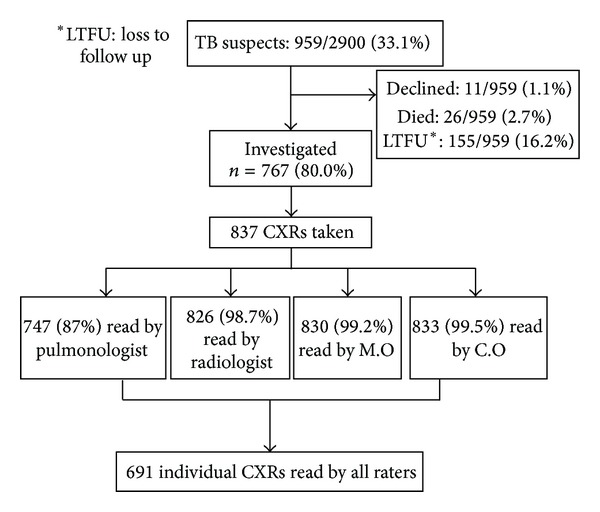
Profile of TB suspects and chest radiographs read per rater.

TB suspects comprised 33% of those enrolled (959/2900). Of these, 767 (80%) consented to CVW admission for additional investigations and 837 chest radiographs were taken (Figure 1).

There were similar numbers of male (51.5%) and female (49.5%) suspects. Some of the prevalent comorbid conditions among suspects included HIV exposure, nearly one-fifth (17.3%), HIV infection (4.4%), and malnutrition (46.0%).

Close to half of investigated infants, 355/767 (46.3%) had been hospitalized with severe lower respiratory tract infections such as bronchiolitis and pneumonia. Prevalence of acute and chronic malnutrition was as follows: wasting (WHZ < −2) *n* = 167/767 (21.7%), stunting (HAZ < −2) 82/767 (10.2%), and underweight (WAZ < −2) 104/767 (13.6%).

Agreement on CXR reading was low overall. For abnormalities consistent with TB (Table 1), poor to moderate agreement was observed across all rater pairs: pulmonologist-radiologist (*k* = 0.24 (95% CI: 0.15–0.34)), pulmonologist-medical officer (*k* = 0.21 (95% CI: 0.13–0.29)), and radiologist-medical officer (*k* = 0.18 (95% CI: 0.10–0.26)).

Agreement among all raters on the diagnosis of lymphadenopathy was moderate, with a multirater weighted *k* = 0.26 (Table 2). On any abnormality on the radiograph, agreement was moderate between expert readers (*k* = 0.28 (95% CI: 0.19–0.37)) and between expert readers and M.O. [Pulmonologist versus medical officer (*k* = 0.23 (95% CI: 0.15–0.31))] and [Radiologist versus Medical Officer (*k* = 0.22 (95% CI: 0.14–0.30))], respectively (Table 4). Poor agreement was registered across all other rater pairs.

There was similar propensity to rate categories between the expert pairs (*P* = 0.14) and between the radiologist and the clinical officer (*P* = 0.24).

Regarding quality of radiographs, little or no agreement was registered across all rater pairs.

The best kappa score was 0.07 (0.03–0.10), observed between the expert pairs (Table 5).

There were eight definite and 40 probable TB cases in the study. One radiograph of a definite TB case was not reviewed by all four raters. The sensitivity and specificity of raters (Table 3) were determined using definite TB cases as the gold standard (*n* = 8). The clinical and medical officers detected the largest proportion of definite TB, while specificity was highest for expert raters.

Due to the small number of definite cases, sensitivity was imprecisely measured. The same table shows low positive predictive values (PPV) (<4.0% for all raters) and high negative predictive values (>99.0% for all raters) for the chest radiograph.

**Figure 2:**
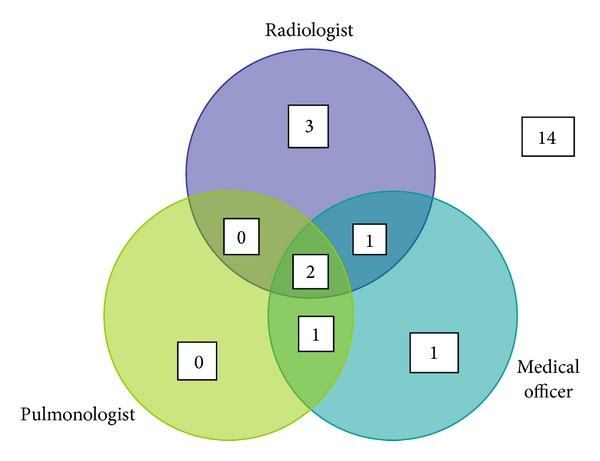
Venn diagram showing radiographs of probable TB cases reviewed by all raters (*n* = 28) and classified as “abnormal likely TB.”

Of 28/40 (70%) probable TB cases whose chest radiographs were read by all raters, experts agreed on only two as being consistent with TB. Such would be the stringent case definition applied in infant TB vaccine trials [14], where only radiographs in which experts agreed (Figure 2) would count as a non-microbiologically confirmed (probable) TB endpoint.

## 4. Discussion

Overall, we observed poor to moderate agreement between experts and between expert and nonexpert pairs. This was consistent across the rater's summary opinion of radiograph as well as on specific pathology such as lymphadenopathy. Our findings fit in with other studies comparing interrater agreement on lymphadenopathy for TB diagnosis [9]. Additionally, we demonstrate that agreement is also poor for the composite assessment of the radiograph, beyond individual radiographic abnormalities. This undermines the reliability of the chest radiograph for TB diagnosis in infant vaccine trials, as well as possibly for clinical diagnosis and patient care.

We suggest several contributing factors. In an infant cohort study with active case finding, suspects are likely to be picked up and investigated before advanced disease with well defined radiographic features is evident. Early tuberculosis is likely associated with greater diagnostic uncertainty and risk of misclassification.

Reference [15]. Interrater agreement studies in a clinical setting where TB suspects may present with more advanced disease are needed to confirm this.

A study of two large infant cohorts in South Africa showed frequent discordance between radiological and microbiological features of TB [2, 16]. The absence of clear, defined radiological abnormalities that correlate well with microbiologically confirmed disease contributes to lack of a reproducible, standardized criteria that raters can use with certainty to evaluate radiographs.

In radiographs of young children, mediastinal abnormalities are difficult to assess and interpret [17] particularly in inexperienced readers, in this case, the C.O and M.O. However, even experienced readers have been found to have poor agreement on lymphadenopathy on chest radiographs [9].

Previous studies had smaller sample sizes for assessment of interrater variability [8, 9]. One of the strengths of this study is the inclusion of a large sample of radiographs for evaluation, from young children with a broad range of respiratory illness and comorbidities. The varying levels of raters' expertise represent the general clinical care structure from primary level to specialized referral hospitals. The findings are therefore applicable to a broad range of settings.

The study had limitations. It was not possible to obtain consensus from expert readers on radiographs on which they differed. This would have increased agreement scores and validity of the chest radiograph as a diagnostic tool. It would also have elucidated patterns of disagreement in order to refine criteria for identifying pathologies consistent with TB on chest radiographs as has been previously recommended [9].

Obtaining high interrater agreement for pediatric chest radiographs in acute pediatric respiratory illness is difficult. Pneumococcal vaccine trials have succeeded in this for opacities consistent with pneumonia on pediatric chest radiographs [18]. Unfortunately, it has been difficult to replicate this success in TB vaccine trials. Conventionally, TB vaccine trial efficacy sample sizes are calculated based on composite endpoints. These include bacteriological and nonbacteriological criteria; as it is expected, microbiologically confirmed cases will contribute to a limited number of endpoints. To increase the number of endpoints and thus reduce sample size requirements, nonbacteriologically confirmed endpoints that rely on chest radiograph findings are included. The latter is defined as radiographic findings compatible with tuberculosis identified independently by two experts [15, 19]. This approach has some limitations. We found that experts agreed only on five of 35 radiographs, as being consistent with TB. Of these, three were bacteriologically confirmed; therefore, the chest radiograph contributed only to two additional endpoints. Poor agreement and high variability in interpreting pediatric CXRs for TB diagnosis among experts increase the probability of misclassifying true disease status and thus underestimating vaccine efficacy [20]. C.Os and M.Os picked up majority of the cases that were later bacteriologically confirmed. This would seem like a positive outcome, given that they work as primary health care providers and would therefore accelerate diagnosis and treatment of infants with TB. However, the limited number of definite cases results in imprecise estimates of sensitivity and should be cautiously interpreted. The high sensitivity also trades off on specificity and could result in unnecessary TB treatment.

Prevailing disease rates influence predictive values. Among infants, the TB incidence of [1.12% (0.54%–2.36%)] would be considered high; however, low PPV relative to sensitivity is attributed to low disease rates. While the NPV was high, where prevalence is not much above 1%, a noninformative test may have a NPV close to 100%.

## 5. Conclusion

Poor agreement and high variability in classifying pediatric radiographs underscores need caution in diagnosing TB in clinical settings where bacteriological confirmation is unavailable, as in most resource limited settings. It further demonstrates that addition of radiographic, nonbacteriologically confirmed endpoints will be of low benefit in decreasing sample sizes for TB vaccine trials.

## Figures and Tables

**(a):** 

Clinician	Medical officer	Radiologist									
		Normal			Abnormal likely TB			Abnormal unlikely TB			Total
		Pulmonologist								
		Normal	Abnormal likely TB	Abnormal unlikely TB	Normal	Abnormal likely TB	Abnormal unlikely TB	Normal	Abnormal likely TB	Abnormal unlikely TB	
Normal	Normal	345	5	30	12	0	3	18	0	4	417
	Abnormal likely TB	5	1	3	1	0	1	0	0	2	13
	Abnormal unlikely TB	83	1	20	3	1	5	10	1	6	130

Abnormal likely TB	Normal	12	1	1	0	0	1	0	0	0	15
	Abnormal likely TB	1	0	0	1	3	0	0	1	0	6
	Abnormal unlikely TB	4	1	0	1	1	1	2	0	0	10

	Normal	51	1	4	3	0	2	4	2	1	68
Abnormal unlikely TB	Abnormal likely TB	4	1	0	1	1	2	0	0	0	9
	Abnormal unlikely	10	0	2	2	1	1	4	2	1	23

Total		515	11	60	24	7	16	38	6	14	691

**(b):** 

Outcome	Kappa (95% CI)	*P* value
TB classification category		
Abnormal	0.177 (0.124–0.237)	<0.001
Abnormal likely TB	0.193 (0.095–0.330)	<0.001
Abnormal unlikely TB	0.065 (0.026–0.125)	<0.001

Generalized/weighted kappa	0.136 (95% CI: 0.100–0.176 )	<0.001

**Table 2:** Overall agreement amongst all raters on lymphadenopathy and quality of CXR.

Rating	Kappa (95% CI)	*P* value
Lymphadenopathy		
Present	0.27 (0.129–0.448)	<0.001
Absent	0.29 (0.163–0.430)	<0.001
Equivocal	0.17 (0.044–0.423)	<0.001
Overall weighted kappa	0.26 (0.182–0.355)	<0.001
Quality of radiographs		
Optimal	−0.1324	0.999
Suboptimal	−0.1674	0.999
Unreadable	−0.0184	0.8822
Overall weighted kappa	−0.1452	0.998

**Table 3:** Sensitivity and specificity: culture confirmed TB versus abnormal likely TB diagnosis on radiograph.

Reader/rater	Culture results		Sensitivity (95% CI)	Specificity (95% CI)	Positive predictive values∗	Negative predictive values∗
	+ve (*n* = 8)	−ve (*n* = 683)				
Clinician						
Positive	5	126	62.5% (24.5%–91.5%)	81.6% (78.4%–84.4%)	3.8% (1.41%–9.13%)	99.5% (98.3%–99.9%)
Negative	3	557				
Medical officer						
Positive	6	185	75.0% (34.9%–96.8%)	72.9% (69.4%–76.2%)	3.14% (1.28%–7.03%)	99.6% (98.4%–99.9%)
Negative	2	498				
Radiologist					3.96% (1.23%–10.0%)	99.3% (98.1–99.8%)
Positive	4	101	50.0% (15.7%–84.3%)	85.2% (82.3%–87.8%)		
Negative	4	582				
Pulmonologist						
Negative	4	110	50.0% (15.7%–84.3%)	83.9% (80.9%–86.6%)	3.5% (1.13%–9.27%)	99.5% (98.1%–99.8%)
Positive	4	573				

∗Disease incidence 1.12% (0.54–2.36).

**Table 4:** Agreement on any abnormality on chest radiograph [(+) abnormal/(−) normal].

*n* = 691						
Reader	−/−	−/+	+/+	+/−	Kappa	McNemar's test
Radiologist versus pulmonologist	515	62	43	71	0.28 (0.19–0.37)	0.44
Pulmonologist versus M.O	445	132	59	55	0.23 (0.15–0.31)	<0.0001
Radiologist versus M.O	450	136	55	50	0.22 (0.14–0.30)	<0.0001
Pulmonologist versus C.O	477	100	31	83	0.10 (0.01–0.18)	0.21
Radiologist versus C.O	493	93	38	67	0.18 (0.10–0.27)	0.04
C.O versus M.O	417	143	48	83	0.10 (0.02–0.17)	<0.0001

**Table 5:** Interrater agreement on quality of chest radiographs.

*n* = 633						
Reader	Optimal-optimal	Optimal-suboptimal	Suboptimal-suboptimal	Suboptimal-optimal	Kappa	McNemar test
Pulmonologist versus radiologist	29	295	301	7	0.07 (0.03–0.10)	<0.0001
M.O versus pulmonologist	10	37	271	314	−0.09 (−0.13–−0.05)	<0.0001
M.O versus radiologist	2	45	551	34	−0.02 (−0.09–0.05)	0.22
C.O versus pulmonologist	7	5	303	317	0.005 (−0.02–0.03)	<0.0001
C.O versus radiologist	2	12	586	34	0.06 (−0.05–0.16)	0.0003
C.O versus M.O	0	12	573	47	−0.03 (−0.05–−0.02)	<0.0001
